# Infection prevention and control practices, policy adherence and knowledge of healthcare workers at COVID-19 treatment centres in Nigeria

**DOI:** 10.4102/ajlm.v14i1.2878

**Published:** 2025-12-12

**Authors:** Adesola Olalekan, Bamidele Iwalokun, Marcellinus Aguwa, Abosede Adegbite, Barakat Bello, Sunday Adesola, Olusola Ojurongbe, Olayinka Ogunleye, Taiwo Ojurongbe

**Affiliations:** 1 Department of Medical Laboratory Science, Faculty of Basic Medical Sciences, College of Medicine, University of Lagos, Lagos, Nigeria; 2 Department of Molecular Biology and Biotechnology, Nigerian Institute of Medical Research, Lagos, Nigeria; 3 Department of Clinical Psychology, Federal Neuro-Psychiatric Hospital, Lagos, Nigeria; 4 Department of Medicine, Federal Neuro-Psychiatric Hospital, Lagos, Nigeria; 5 Infectious Diseases Hospital, Lagos, Nigeria; 6 Center for Emerging and Re-emerging Infectious Diseases, Ladoke Akintola University of Technology, Oyo, Nigeria; 7 Department of Pharmacology, Therapeutics and Toxicology, College of Medicine, Lagos State University, Lagos, Nigeria; 8 Department of Statistics, Osun State University Osogbo, Osun, Nigeria

**Keywords:** infection prevention and control, healthcare workers, coronavirus disease 2019, facility policies, pandemic preparedness, knowledge assessment

## Abstract

**Background:**

Infection prevention and control (IPC) practices are crucial for protecting patients and healthcare workers (HCWs), especially during the coronavirus disease 2019 (COVID-19) pandemic. This crisis has underscored the importance of IPC strategies in understanding health system readiness and strengthening preparedness for future pandemics.

**Objective:**

This study investigated healthcare personnel’s IPC knowledge, adherence to safety policies, and implementation of IPC procedures in COVID-19 treatment centres across Nigeria.

**Methods:**

A cross-sectional, multicentre study was conducted among 113 respondents, that is, 57 HCWs and 56 volunteers, from 23 June 2020 to 15 March 2021. An electronic questionnaire adapted from validated instruments was used.

**Results:**

Out of 113 respondents, 69 (61%) demonstrated good IPC practices, with high adherence (*n* = 105, 92.9%) to face mask usage and hand hygiene. Only 50 (44.2%) reported receiving basic training on IPC. Although personal protective equipment (PPE) was available, 25% did not consistently wear full PPE when attending to COVID-19 patients. Most HCWs (105; 93%) opposed testing patients without consent, and 100 (88.5%) affirmed the availability of standardised IPC protocols. No significant association was observed between age, gender, years of experience, and IPC compliance (*p*-values: 0.097, 0.287, and 0.699). Interestingly, 33 (29.2%) HCWs with less than 10 years of experience exhibited better IPC practices. Facility policies such as confidentiality and non-discrimination were mostly upheld, with 90 (79.6%) participants agreeing that discriminatory practices should have consequences.

**Conclusion:**

While face mask use and hand hygiene compliance were high, gaps remained in IPC training and consistent use of full PPE. Strengthening training, IPC knowledge, policy standardisation, and resource equity is important for stronger IPC compliance during health emergencies.

**What this study adds:**

The study identified key factors supporting future pandemic preparedness by examining the control and preventive strategies implemented at various CTCs in Nigeria. It also emphasised the need for standardised policies, which are essential for building resilient healthcare systems during public health crises.

## Introduction

Infection prevention and control (IPC) is a holistic approach aimed at curbing and halting the transmission of infectious agents and the diseases they cause among healthcare workers (HCWs), patients, their relatives, and the broader community. When IPC measures are not implemented properly, they can lead to a decline in the quality of healthcare, increased harm, and the spread of diseases. Healthcare workers and other staff in health institutions are key stakeholders in the implementation of IPC activities, such as hand hygiene, use of personal protective equipment (PPE), injection safety, and adherence to World Health Organization (WHO) standard guidelines,^1^ especially during public health emergencies such as the COVID-19 pandemic. Infection prevention and control practices were compromised during the COVID-19 pandemic because of a shortage of IPC experts and volunteers to support personal hygiene and facility-level infection prevention efforts.^2^

The United States Centers for Disease Control and Prevention recommended strict IPC guidelines for COVID-19 to help limit its transmission. These guidelines included the use of visible signage (e.g. signs and posters) at entrances and other strategic locations such as waiting areas, elevators and cafeterias. The United States Centers for Disease Control and Prevention also emphasised using respiratory protection, such as respirators, well-fitting face masks, or cloth masks to cover the mouth and nose, thereby reducing the spread of respiratory secretions during breathing, sneezing, talking and coughing. The universal use of PPE by healthcare personnel was encouraged strongly through distancing and avoiding crowded areas. In addition, the United States Centers for Disease Control and Prevention recommended optimising engineering controls to reduce exposure risks. For example, setting up physical barriers in reception and triage areas and establishing designated routes to direct symptomatic patients through waiting rooms and triage areas. Dedicated medical equipment was advised to minimise cross-contamination when managing patients with confirmed or suspected severe acute respiratory syndrome coronavirus 2 infection.^3^ One of the improvisations of the COVID-19 era in Africa was the provision of Veronica buckets in different locations in the COVID-19 treatment centres (CTCs). This ensured access to running water for hand washing, aligning with hand hygiene principles in IPC protocols.^4^

Furthermore, the WHO has underscored consistently the necessity for well-designed and effectively executed IPC programmes to curb the transmission of the virus both domestically and internationally.^5^ Infection prevention and control encompasses a set of practices and procedures that must be implemented in healthcare settings to prevent the transmission of infections between patients and HCWs.^6^ Effective IPC also maintains a safe hospital environment and safeguards against community transmission of infectious illnesses during outbreaks.^7^

Successful pandemic control, such as during COVID-19, requires IPC strategies guided by well-established policies.^8^ However, poor compliance with IPC measures in low- and middle-income countries, especially in Africa and Asia, has been a significant challenge in managing the COVID-19 pandemic.^9,10,11,12^

During the COVID-19 pandemic, the implementation of IPC measures varied between low- and middle-income countries and high-income countries. Differences were observed in compliance strategies, available infrastructure, and the level of facility engagement.

Nigeria, one of Africa’s most populous countries, was globally recognised for its swift and effective containment of the Ebola outbreak in 2014.^13^ However, its capacity and strategy to control the COVID-19 pandemic through effective IPC implementation in treatment centres remains insufficiently documented. A key factor in strengthening future pandemic preparedness is the understanding of the infection control and prevention strategies that were employed in various CTCs in Nigeria. This includes evaluating adherence to IPC policies and assessing the awareness of COVID-19-related protocols among HCWs and volunteers. Such data can serve as a foundation for developing more robust preparedness plans for future emerging infectious diseases.

Therefore, this study aims to evaluate the infection prevention strategies used by HCWs and volunteers in CTCs in Nigeria, along with their knowledge of disease control, confidentiality practices and adherence to facility policies.

## Methods

### Ethical considerations

Ethical approval for the survey was obtained from the Hospital Research Ethics Committee of the College of Medicine, University of Lagos (Ethics approval number: CMUL/HREC/05/20/738). The purpose of the research was outlined clearly in the electronic questionnaire, which included a statement of consent for participation. No names or other identification information was collected, and a statement was provided on the respondents’ rights and privacy. Participants were only able to complete the questionnaire after indicating their agreement to the ethical and consent declarations

### Study design and population

**FIGURE 1:**
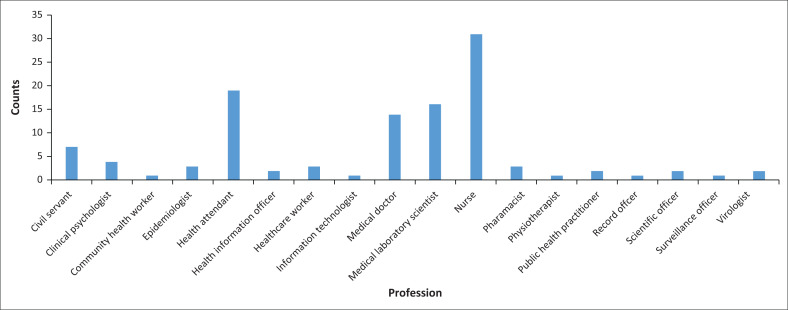
Distribution of healthcare workers and volunteers who participated in the study, Nigeria, 23 June 2020 to 15 March 2021.

This cross-sectional, questionnaire-based multicentre study involved 113 workers, comprising 57 HCWs and 56 volunteers from various CTCs across Nigeria, with the majority based in Lagos (82; 75%), the epicentre of the outbreak in the country. The study was conducted between 23 June 2020 and 15 March 2021. Other states represented included Ogun (7; 6.2%), Oyo and the Federal Capital Territory Abuja (4; 3.5% each), Bayelsa (3; 2.6%), Katsina, Kebbi, Osun, and Rivers (2; 1.8% each), and Abia, Borno, Ebonyi, Kwara, and Zamfara (1; 0.9% each) (Table 1). A variety of healthcare professionals and support personnel working in CTCs participated in the study, including nurses (31; 27.43%), health attendants (19; 16.81%), medical laboratory scientists (16; 14.16%), medical doctors (14; 12.39%), and pharmacists (3; 2.65%), along with others (30; 26.56%) (detailed in Figure 1). All participants provided consent and completed the electronic questionnaire-based survey, which was disseminated via online platforms, including email and WhatsApp group chats. Nurses and ward attendants were involved in patient care; medical laboratory scientists performed patient investigations, including nasal swabbing for severe acute respiratory syndrome coronavirus 2 testing and confirmation; medical doctors also provided patient care; pharmacists were responsible for dispensing drugs; and other volunteers contributed their professional skills in various roles at the CTCs. In this study, a volunteer is defined as a respondent who is not employed full-time in a COVID-19-related hospital, but who volunteered at a CTC (Table 2).

**TABLE 1:** Frequency of the participants per state in Nigeria, 23 June 2020 to 15 March 2021.

State	*n*	%
Abia	1	0.9
Abuja	4	3.5
Bayelsa	3	2.6
Borno	1	0.9
Ebonyi	1	0.9
Katsina	2	1.8
Kebbi	2	1.8
Kwara	1	0.8
Lagos	82	72.5
Ogun	7	6.2
Osun	2	1.8
Oyo	4	3.5
Rivers	2	1.8
Zamfara	1	0.9

**Total**	**113**	**100**

**TABLE 2:** Socio-demographic analysis of the respondents, Nigeria, 23 June 2020 to 15 March 2021.

Variable	Categories	*n*	%
Age group (years)	≤ 30	14	12.4
	31–50	79	69.9
	≥ 51	20	17.7
Years of experience	≤ 10	51	45.1
	10–20	41	36.3
	≥ 21	21	18.6
Religion	Christianity	74	65.5
	Islam	39	34.5
Is your facility involved in COVID-19 treatment, or are you a volunteer at a COVID-19 treatment centre?	I am not working in COVID-19 related hospital	9	8.0
	No, I am a volunteer at a COVID-19 treatment centre	56	49.6
	Yes, my centre is involved in COVID-19 treatment.	48	42.5
Cases of suspected COVID-19 patients you provided with care or services?	Nil	9	8.0
	1–100	43	38.1
	101–500	32	28.3
	501 and above	29	25.7
How many months did you work in the COVID-19 facility?	Nil	5	4.4
	1–3	44	38.9
	4–6	52	46.0
	Above 6	12	10.6

COVID-19, coronavirus disease 2019.

### Questionnaire

The electronic questionnaire used to collect information from respondents was a modified version of the validated instrument for measuring HIV-related stigma and discrimination among health facility staff. This comprehensive questionnaire, originally developed by the United States Agency for International Development, the United States President’s Emergency Plan for AIDS Relief, and the Health Policy Project, was adapted for this study.^14^ In addition, excerpts from the WHO guidelines on IPC strategies for COVID-19^5^ were incorporated to assess that COVID-19 IPC processes were carried out across various facilities.

The self-administered electronic survey comprised two sections. The first section of the questionnaire collected demographic information, including gender, age (as of the respondent’s last birthday), profession, location of COVID-19 service delivery, and years of experience. The second section evaluated respondents’ knowledge, facility policies, and infection control practices using a three-point Likert scale with response options: Yes, No, or Not Applicable.

### Data analysis

The database was automatically generated in an Excel spreadsheet, then collated and exported to Statistical Package for the Social Science version 27 (IBM Corp., Armonk, New York, United States) for descriptive and comparative analyses. The chi-square test was employed to compare different categories of respondents involved in the study and to analyse prevention and control activities in the CTCs. Those who had a score below the mean for the IPC practice variables (avoiding physical contact, wearing of face masks, wearing of gloves during all aspects of patient care, implementing any special IPC measures for patients with COVID-19 that are not used for other patients, and wearing full PPE), when pooled together, were classified as having poor or inadequate use of protective measures; and those who scored greater than or equal to the mean score were classified as having good or adequate practices.

## Results

### Demographics and general characteristics of the respondents

The total number of participants was 113, of whom 79 (69.9%) were between 31 and 50 years of age, and 51 (45.1%) had less than 10 years of experience in infectious disease-related work. Approximately half (56; 49.6%) of the respondents who worked in the CTCs at the time of the study were volunteers (Table 2). There was no significant association between the socio-demographic variables and the IPC practices by all the respondents (Table 3). The distribution of the category of HCWs and volunteers involved in this study is indicated in Figure 1. Cross-tabulation between the professional category of respondents and the category of training indicated that some volunteers had prior training in IPC, as they were healthcare professionals who volunteered to work in the CTCs during the pandemic. These individuals were not originally part of the full-time staff of CTC (Table 4).

**TABLE 3:** Association between socio-demographic and infection control practice among the respondents, Nigeria, 23 June 2020 to 15 March 2021.

Variable	Categories	Poor practice		Good practice		Total		Pearson (*χ*^2^)	*df*	*p*
		*n*	%	*n*	%	*n*	%			
Age group (years)	-	-	-	-	-	-	-	4.666	2	0.097
	≤ 30	2	1.8	12	10.6	14	12.4	-	-	-
	31–50	35	31.0	44	38.9	79	69.9	-	-	-
	≥ 51	7	6.2	13	11.5	20	17.7	-	-	-
Gender	-	-	-	-	-	-	-	1.135	1	0.287
	Female	21	18.6	40	35.4	61	54.0	-	-	-
	Male	23	20.4	29	25.7	52	46.0	-	-	-
Years of experience	-	-	-	-	-	-	-	0.716	2	0.699
	≤ 10	18	15.9	33	29.2	51	45.1	-	-	-
	10–20	18	15.9	23	20.4	41	36.3	-	-	-
	≥ 21	8	7.1	13	11.5	21	18.6	-	-	-
Religion	-	-	-	-	-	-	-	0.006	1	0.940
	Christianity	29	25.7	45	39.8	74	65.5	-	-	-
	Islam	15	13.3	24	21.2	39	34.5	-	-	-

*df*, degrees of freedom; χ^2^, Chi-square.

**TABLE 4:** Classification of training by profession, Nigeria, 23 June 2020 to 15 March 2021.

Profession	Classification of Training										
	A	A, B	A, C	A, B, C	B	D	C, D	A, C, D	A, B, C, D	B, D	Total
Civil servant	5	0	0	0	1	0	0	1	0	0	**7**
Clinical psychologist	0	0	0	0	0	0	0	0	3	1	**4**
Community health worker	1	0	0	0	0	0	0	0	0	0	**1**
Epidemiologist	1	0	0	0	0	0	0	0	2	0	**3**
Health attendant	15	0	0	0	0	0	0	1	3	0	**19**
Health information officer	0	0	0	0	0	0	0	0	2	0	**2**
Healthcare worker	1	0	0	1	0	0	0	0	1	0	**3**
Information technologist	0	0	1	0	0	0	0	0	0	0	**1**
Medical doctor	0	1	2	0	0	0	0	1	10	0	**14**
Medical laboratory scientist	3	0	0	0	0	1	1	2	9	0	**16**
Nursing	10	0	2	0	0	0	1	3	15	0	**31**
Pharmacist	2	0	0	0	0	0	0	1	0	0	**3**
Physiotherapy	0	0	0	0	0	0	0	0	1	0	**1**
Public health practitioner	0	0	0	0	0	0	1	0	1	0	**2**
Record officer	0	0	0	0	0	1	0	0	0	0	**1**
Scientific officer	0	0	0	0	0	0	0	0	2	0	**2**
Surveillance officer	0	0	0	0	0	0	1	0	0	0	**1**
Virologist	0	0	0	0	0	0	0	1	1	0	**2**

**Total**	**38**	**1**	**5**	**1**	**1**	**2**	**4**	**10**	**50**	**1**	**113**

Note: A: Infection prevention and control of COVID-19; B: Key population stigma and discrimination; C: Patients’ informed consent, privacy, and confidentiality; D: Patients’ confidentiality.

COVID-19, coronavirus disease 2019.

### Knowledge of infection prevention and control strategies

Most (44.2%) of the respondents had received basic training on patient confidentiality, IPC of COVID-19, privacy, patient informed consent, discrimination and key population stigma. The other category of respondents had received training in IPC for COVID-19 (33.6%), as shown in Table 4. Table 5 highlights the different training categories to which respondents were exposed. Key population stigma and discrimination, particularly in the context of COVID-19 care, refer to the negative attitudes and beliefs directed towards individuals with COVID-19, and discrimination as denial of care or unequal treatment. Knowledge of this is essential to promote equity, trust and adherence to ethical standards in patient management.^14^

**TABLE 5:** Previous training by the participants, Nigeria, 23 June 2020 to 15 March 2021.

Previous training received by the respondents	*n*	%
Infection prevention and control of COVID-19	38	33.6
Infection prevention and control of COVID-19, Key population stigma and discrimination	1	0.9
Infection prevention and control of COVID-19, Patients’ informed consent, privacy, and confidentiality	5	4.4
Infection prevention and control of COVID-19, Patients’ informed consent, privacy, and confidentiality, Key population stigma and discrimination	1	0.9
Key population stigma and discrimination	1	0.9
Patients’ confidentiality	2	1.8
Patients’ confidentiality, Infection prevention and control of COVID-19	4	3.5
Patients’ confidentiality, Infection prevention and control of COVID-19, Patients’ informed consent, privacy, and confidentiality	10	8.8
Patients’ confidentiality, Infection prevention and control of COVID-19, Patients’ informed consent, privacy, and confidentiality, Key population stigma and discrimination	50	44.2

COVID-19, coronavirus disease 2019.

### Infection control practices by the healthcare workers in the COVID-19 treatment centres

**FIGURE 2:**
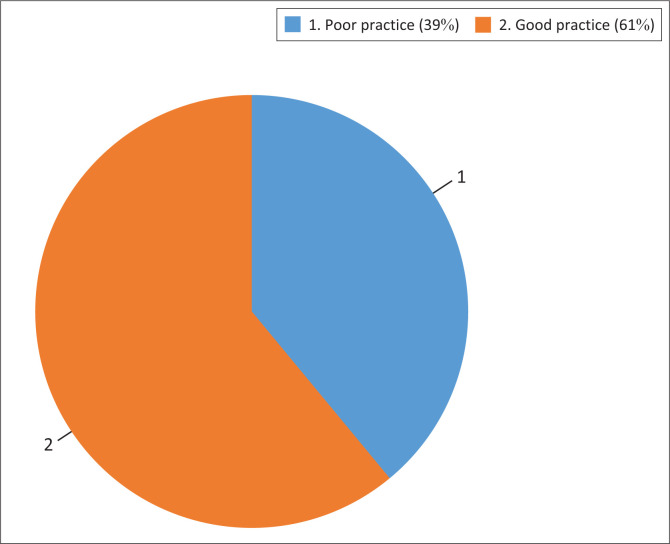
Infection control practices among the respondents, Nigeria, 23 June 2020 to 15 March 2021.

Infection control practices that most HCWs could apply vary between good practices (61%) and those IPC methods that were poorly executed (39%) (Figure 2). The application of alcohol-based hand sanitisers, the use of face masks, and hand washing with soap and water were the most common practices among the HCWs in the CTCs (Table 6). Most HCWs did not wear complete PPE during the care and management of patients at the centres. Of the HCWs in these centres, 88 (77.9%) use special infection-control measures for patients infected with COVID-19, which they normally do not use with other patients not infected with COVID-19 (Table 7). In addition, 75% of healthcare workers reported wearing full PPE while working with COVID-19-confirmed patients at the treatment centres.

**TABLE 6:** Distribution of infection prevention and control practices (*N* = 113), Nigeria, 23 June 2020 to 15 March 2021.

Variable	Categories	*n*	%
Hand hygiene	Good	15	13.3
	Very good	98	86.7
Respiratory etiquette (use of face mask)	Good	16	14.2
	Very good	97	85.8
Personal protective equipment	Good	20	17.7
	Not at all	5	4.4
	Very good	88	77.9
Safe injection practices and sharps management	Good	18	15.9
	Not at all	7	6.2
	Very good	88	77.9
Cleaning and disinfection of patient-care equipment	Good	23	20.4
	Not at all	5	4.4
	Very good	85	75.2
Environmental cleaning and safe handling and cleaning of soiled linen, as well as waste management	Good	28	24.8
	Not at all	5	4.4
	Very good	80	70.8
Do you use water and soap to wash your hands?	No	3	2.7
	Yes	110	97.3
Do you use a single-use towel?	No	33	29.2
	Yes	80	70.8
Do you use alcohol-based rub for hand hygiene?	No	2	1.8
	Yes	111	98.2
Do you use a medical mask all the time in your facility?	No	2	1.8
	Yes	111	98.2
Do you always clean your hands before and after the wearing of the personal protective equipment?	No	1	0.9
	Not applicable	4	3.5
	Yes	108	95.6

COVID-19, coronavirus disease 2019.

**TABLE 7:** Infection prevention control practices at the COVID-19 treatment centres, Nigeria, 23 June 2020 to 15 March 2021.

Characteristics	Indifferent		Most likely		Not likely		Total	
	*n*	%	*n*	%	*n*	%	*n*	%
Avoid physical contact with COVID-19 patients	14	12.4	70	61.9	29	25.7	113	100.0
Wearing face mask	4	3.5	105	92.9	4	3.5	113	99.9
Wear gloves during all aspects of the patient’s care	7	6.2	102	90.3	4	3.5	113	100.0
Use any special infection-control measures with patients infected with COVID-19 that you do not use with other patients	13	11.5	88	77.9	12	10.6	113	100.0
Do you often wear the full personal protective equipment?	18	15.9	75	66.4	20	17.7	113	100.0

COVID-19, coronavirus disease 2019.

### Health policy and regulations in the facility

Basic policies and regulations such as informed consent, discrimination and confidentiality, supply of IPC materials in the facility, and standard hospital protocol in handling patients were assessed among HCWs in these treatment centres. Most of the HCWs (93%) believe that testing individuals without their knowledge (informed consent) is not advisable. In addition, 81.4% believed that there are sufficient supplies of IPC materials and PPE in the facilities, while 88.5% feel that the standard operating procedures guiding their daily operations at the centres are accessible (Table 8). Interestingly, 15.9% of the HCWs in this study are indifferent to issues of patient discrimination and disclosure of their patients’ status (confidentiality) while working at the facility (Table 8).

**TABLE 8:** Health policy and regulations in the facility, Nigeria, 23 June 2020 to 15 March 2021.

Characteristics	Agree		Disagree		Don’t know		Total	
	*n*	%	*n*	%	*n*	%	*n*	%
Do you think it is advisable to test a patient for COVID-19 infection without their knowledge?	8	7.0	105	93.0	0	0	113	100
I will get into trouble at work if I discriminate against COVID-19 patients and disclose their status.	90	79.6	5	4.4	18	15.9	113	100
There are adequate supplies in my health facility that reduce my risk of becoming infected with COVID-19.	92	81.4	21	18.6	0	0	113	100
There are standardised procedures/protocols in my health facility that reduce my risk of becoming infected with COVID-19.	100	88.5	13	11.5	0	0	113	100

COVID-19, coronavirus disease 2019.

## Discussion

Our study observed that 44.2% of respondents had received basic training on IPC related to COVID-19, including hand hygiene, use of PPE, patient informed consent, privacy, confidentiality, and issues surrounding key population stigma and discrimination. The most common IPC practices among HCWs at the treatment centres were the use of face masks, handwashing with soap and water, and the application of alcohol-based hand sanitisers. In addition, 75% of respondents reported wearing full PPE while attending to patients confirmed to have COVID-19. Notably, 93% of HCWs believed that testing individuals without their knowledge or consent was inappropriate.

This study highlighted the knowledge and practices of IPC among HCWs and volunteers who worked in CTCs in Nigeria during the 2020–2022 pandemic, primarily in Lagos State (Table 1). The study also examined their adherence to established policies in these facilities.

The WHO emphasises that IPC is vital for limiting disease transmission and safeguarding both HCWs and the wider community. The attitudes, knowledge and practices of HCWs regarding IPC play a crucial role in ensuring patient safety and improving treatment outcomes.

In this study, years of work experience, age, and gender are not associated with the infection control practices among the respondents (*p* > 0.05). Of the 113 participants, 61 (54%) were male and 52 (46%) were female. Of the women, 40 (57.8%) demonstrated good IPC, which is lower than the 70.4% reported among 301 female participants in a similar study conducted in Kaduna, Nigeria.^15^ These differences may be due to variations in training, occupational roles, and hygiene practices. In a study from Ethiopia, male HCWs were three times more likely to demonstrate good IPC,^16^ a trend also seen in China, and attributed to their lighter workloads and differing occupational roles.^17^

Our study observed that HCWs with fewer years of work experience (≤ 10 years) demonstrated better IPC in 33 out of 69 (47.8%) cases, which may be attributed to recent updated training methods, adherence to protocols owing to fear of consequences, technological adaptability, supervisory pressure and heightened awareness. In contrast, a study conducted in Ethiopia in 2021 found that HCWs with longer years of experience had good IPC practices compared to those with less than 5 years of experience.^16^ Similarly, in Pakistan, logistic regression analysis showed that experienced (> 5 years) HCWs were more likely to adhere to precautionary practices, probably because of their skills and experience in managing with public health emergencies.^18^

In our study, healthcare workers and volunteers aged between 31 and 50 demonstrated good IPC practices in 44 (38.9%) cases. This contrasts with findings from South Africa, where younger HCWs achieved higher knowledge scores than their older counterparts, possibly because of their broader use of digital information sources.^19^ However, another study suggests that older HCWs may perform better because of their accumulated experience.^18^

Only 50 (44.2%) of our study participants had basic knowledge of IPC, confidentiality, and consent, in contrast with studies in Ethiopia (88.2%) and Afghanistan (85%), which reported a higher level of IPC knowledge.^20,21^ Similarly, a study in Nepal reported 76% adequate IPC knowledge,^22^ while another Nigerian study reported 56%.^23^ These discrepancies may be because of differences in sampling methods or to the scope of the questionnaire items. The relatively low knowledge level in our study may also reflect the small proportion (33%) of respondents who received COVID-19-specific IPC training (Table 4). Comprehensive training and ongoing education should be prioritised.

Our study showed that 61% of HCWs practised good IPC, including avoiding physical contact, wearing of face masks and gloves during all aspects of patient care, implementing special IPC measures for COVID-19 patients, and wearing full PPE for COVID-19, higher than the 41% reported in another Nigerian study^15^ but lower than compliance rates observed in India, Uganda, and Ghana.^24,25,26^

The most common IPC practices included wearing face masks (92.9%), followed by wearing gloves during all aspects of patient care (90.3%) (Table 7). These findings align with a Ugandan study reporting 93% mask use.^26^ However, in Ghana, PPE use was reported to be lower.^27^ In additional, some other studies highlighted poor adherence to mask and glove use, often because of discomfort and PPE shortages.^28,29,30,31^

Most participants in our study reported that IPC supplies were adequately available, reducing their perceived risk of contracting COVID-19. In contrast, a Brazilian study noted significant PPE shortages.^32^ Adequate PPE is essential to prevent HCW infections and reduce community transmission. Other studies similarly reported good supply availability during COVID-19 management.^33,34^

A large majority (93%) of our participants affirmed the availability of standard operating procedures in their facilities, which enhances both patient care and infection prevention. However, Iyal and colleagues attributed low IPC compliance in their study to the lack of COVID-19-specific guidelines.^15^ Meanwhile, a study in Ghana reported low protocol adherence despite the availability of guidelines.^27^ Therefore, making protocols readily accessible and promoting adherence are critical to effective IPC in treatment centres.

Interestingly, 93% of our participants disagreed with testing patients for COVID-19 without their consent. This finding, however, does not align with WHO guidance, which allows for exceptions during public health emergencies under specific legal frameworks.^35^ A United States study revealed public support for clear communication and consent, although mandatory testing was accepted in high-risk settings like schools or hospitals.^36^ The study also assessed perceptions of discrimination, with 79.6% of respondents indicating that there would be consequences for discriminating against patients with COVID-19. This probably reflects awareness of professional ethical standards,^37^ and underscores the role of ethical compliance in building trust and improving patient outcomes. Respect for confidentiality and consent remains fundamental to effective healthcare delivery.

### Limitations of the study

The small number of participants and geographical distribution limit the generalisation of the study. In addition, the study is based on self-reported data and information provided by the participants, which could not be verified. Secondly, the potential influence of social desirability bias might have affected the responses on IPC compliance. Understanding the context of the COVID-19 response, participants may have felt compelled to indicate higher adherence to IPC protocols than what was done. This bias may have led to an exaggerated view of compliance rates. Future studies must validate self-reported data using other methods to ascertain accurately the IPC practices carried out by the respondents. However, despite these limitations, this constraint did not undermine its overall significance and the findings can inform future research and policy development for pandemic preparedness. Lagos State was the epicentre of the COVID-19 pandemic in Nigeria, which accounts for the higher number of respondents from treatment centres in the state. Future research should include representation from all six geopolitical zones to generate findings that can be generalised and inform national policy.

### Conclusion

Generally, our study highlights the infection control practices carried out in the CTCs. Practices such as the use of alcohol-based hand rub, wearing of medical masks, and handwashing with soap and water were the predominant infection prevention measures implemented at the centres. In addition, HCWs demonstrated notable knowledge of COVID-19 and adhered to consent and confidentiality protocols in several treatment facilities across Nigeria during the pandemic. These findings support improved preparedness and response strategies for future emerging or re-emerging infectious diseases in Africa. Continuous training and improved knowledge of IPC, particularly in the proper use of PPE, transmission modes, encouraging adherence, enforcement of sanctions for non-compliance and containment strategies, are essential for strengthening health systems and for protecting HCWs.
